# Collagen implant *versus* gluteus maximus flap for perineal closure after extended abdominoperineal excision: NEAPE randomized clinical trial

**DOI:** 10.1093/bjsopen/zrag079

**Published:** 2026-06-24

**Authors:** Martin Rutegård, Johan Svensson, Jörgen Rutegård, Tero Rautio, Per J Nilsson, Olle Sjöström, Marie-Louise Lydrup, Christoffer Odensten, Andreas Söderström, Michael Dahlberg, Markku M Haapamäki

**Affiliations:** Department of Diagnostics and Intervention, Surgery, Umeå University, Umeå, Sweden; Department of Diagnostics and Intervention, Surgery, Umeå University, Umeå, Sweden; Department of Statistics, Umeå School of Business, Economics and Statistics, Umeå University, Umeå, Sweden; Department of Diagnostics and Intervention, Surgery, Umeå University, Umeå, Sweden; Oulu University Hospital, Medical Research Centre Oulu, Oulu, Finland; Department of Molecular Medicine and Surgery, Karolinska Institutet, Stockholm, Sweden; Department of Diagnostics and Intervention, Surgery, Umeå University, Umeå, Sweden; Department of Surgery, Skåne University Hospital, Malmö, Lund University, Lund, Sweden; Department of Diagnostics and Intervention, Surgery, Umeå University, Umeå, Sweden; Department of Diagnostics and Intervention, Surgery, Umeå University, Umeå, Sweden; Department of Diagnostics and Intervention, Surgery, Umeå University, Umeå, Sweden; Department of Diagnostics and Intervention, Surgery, Umeå University, Umeå, Sweden

**Keywords:** rectal cancer, neoplasm of rectum, extralevator abdominoperineal excision, reconstructive procedures, postoperative disability

## Abstract

**Background:**

Optimal reconstruction of the perineal defect after extended abdominoperineal excision remains uncertain, and randomized comparisons between biological mesh and myocutaneous flaps are lacking. This trial assessed whether an acellular porcine collagen implant (APCI) is non-inferior to a gluteus maximus myocutaneous flap (GMF) for pelvic floor reconstruction.

**Methods:**

NEAPE was a multicentre, randomized, non-inferiority trial conducted in eight Nordic referral centres. Adults undergoing extended abdominoperineal excision for rectal cancer were randomized to reconstruction with an APCI or unilateral GMF. The primary outcome was the proportion of patients underperforming in the timed-stands test at 6 months, defined relative to age- and sex-matched reference values. The non-inferiority margin was −10%.

**Results:**

Eighty-three patients were included in the modified intention-to-treat population (mean(standard deviation) age 68(10) years; 31% female). At 6 months, 71% of patients (25 of 35) in the implant group and 60% of patients (20 of 33) in the flap group underperformed. The risk difference was −11% (one-sided 95% confidence interval −30% to ∞), and non-inferiority was not demonstrated.

**Conclusion:**

The APCI did not demonstrate non-inferiority compared with the GMF. The direction of the effect favoured the flap, with results compatible with worse functional outcomes in the implant group.

**Registration number:**

ClinicalTrials.gov NCT01347697.

## Teaser

This randomized multicentre clinical trial investigated whether an acellular porcine collagen implant result in less functional problems than a gluteus maximus myocutaneous flap for the reconstruction of the pelvic floor after an extended abdominoperineal resection. Comparing the postoperative physical performance of 85 patients with rectal cancer, non-inferiority of the acellular porcine collagen implant could not be demonstrated.

## Introduction

Abdominoperineal excision (APE) is the standard non-restorative procedure for distal rectal adenocarcinoma for oncological or functional reasons^1^. The local recurrence rate after APE has historically been high^2,3^, prompting the development of the extended APE, where the levator musculature is included by a lateral dissection close to the pelvic wall, which forms a cylindrical specimen^1,4,5^. An extended APE results in a large perineal defect. Although primary suture of the subcutaneous fat and skin is possible, reconstruction with biological mesh decreases the risk of a perineal hernia^6^. Other reconstruction techniques have been used to avoid the consequences of foreign material implants^7^. For example, a gluteus maximus myocutaneous flap (GMF) can be used to avoid damage to the abdominal wall, facilitating a standard minimally invasive approach for the oncological procedure, resulting in low local complication rates^8^. However, these patients sometimes have difficulties sitting down without aids and experience reduced physical function^9^. These impairments could potentially be reduced using muscle-sparing flap techniques, which have been studied less^10,11^. Although a recent randomized trial by Kreisel *et al*. indicated that presacral abscess formation was less common after gluteal turnover flaps compared with primary closure, more high-quality prospective trials are needed that compare reconstruction methods after extended APE^12–14^.

This NEAPE trial tested the hypothesis that an acellular porcine-collagen implant (APCI) is not inferior to a GMF for reconstruction of the pelvic floor after extended APE for rectal cancer.

## Methods

### Trial design

**Fig. 1:**
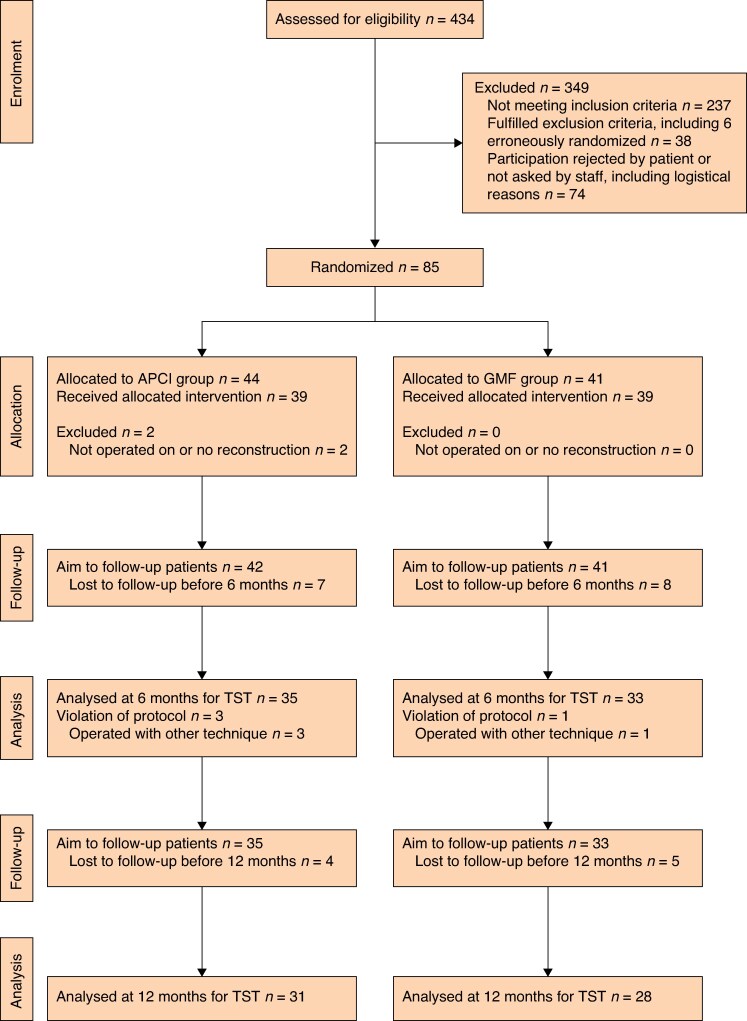
Study flow chart APCI, acellular porcine collagen implant; GMF, gluteus maximus myocutaneous flap; TST, timed-stands test.

This was a randomized, controlled, non-blinded, multicentre non-inferiority trial in eight Nordic referral hospitals with two parallel groups (*Fig. 1*). The study followed the CONSORT guidelines^15^.

The study was approved by the Regional Ethical Review Board at Umeå University, Umeå, Sweden (Protocol no: NEAPE-2010-335-31M) and the Oulu University Hospital Ethical Review board in Oulu, Finland.

The study was registered with ClinicalTrials.gov (NCT01347697) and the protocol, including the analysis plan, was published online at study launch and later in a peer-reviewed publication^16^. The size of the randomization blocks, the number of operated patients per hospital, the CONSORT checklist, and the original study protocol are provided in the *Supplementary material*.

### Patients

Adult patients (age ≥ 18 years) with rectal cancer who were scheduled to receive extended APE and who had a life expectancy beyond 1 year after surgery were eligible for inclusion. Preoperative radiotherapy consisted of short-course radiotherapy (5 × 5 Gy) or long-course radiotherapy (25 × 2 Gy), with or without chemotherapy. The exclusion criteria were the need for sacrectomy, bilateral flap reconstruction, or reconstruction of the vaginal wall. Written informed consent was obtained from all participants^16^.

### Randomization and blinding

Eligible patients were randomized to reconstruction with an APCI (experimental arm) or a GMF (standard arm), either before the extended APE operation or during APE. Patients were block-randomized with an allocation ratio of 1 : 1 with stratification for centre and use of preoperative radiotherapy. The randomization sequence was computer generated, using a random number technique and concealed. The randomization, using permuted and concealed blocks, was done online via a web application (norrlandskirurgi.se). The entire process of registration, randomization, and allocation was completed on the study homepage (www.norrlandskirurgi.se), with coding performed by a system developer independent of the remainder of the study recruitment, conduct, and analysis. This trial was performed without blinding.

### Procedures

All patients received preoperative antibiotics and antithrombotic prophylaxis, according to the local site protocol. Open or minimally invasive techniques were used for the abdominal part of the operation, whereas the perineal part was performed with an open technique with the patient in a prone position. Intraoperative randomization was used for patients where inclusion in the study could not be determined before this point. If the operation could be performed with a safe margin to the tumour without excision of the levator muscles on both sides, primary suture of the defect was often chosen and the patient could not be randomized in the study because no reconstruction was needed. For included (and randomized) patients, the principles of an extended (also known as extralevator) approach were used; that is, the levator muscles were divided at their lateral insertions^8^. Disarticulation of the coccyx and the use of omentoplasty were left to the discretion of the operating surgeon. Any vaginal wall defects were closed with absorbable sutures, and an intra-abdominal drain was placed.

In the experimental arm, a piece of APCI (1.5 mm thick) was tailored to fit the pelvic floor defect. The implant was sutured in place to the remnants of the levator musculature with 2/0 polypropylene thread using interrupted sutures. The wound was drained in two layers with the deep drain adjacent to the implant and the second superficially below the skin. The wound was closed in three layers with interrupted monofilament sutures.

In the control arm, the pelvic floor was reconstructed using a unilateral GMF, as described in the study protocol^16^ and in an earlier publication by Holm *et al*.^8^. The flap was based cranially with the length approximately 1.5 : 1 in proportion to the base. The skin and subcutaneous tissue were incised down to the gluteus maximus, where the fascia was also incised along the whole length of the incision to add mobility to the flap. Approximately one-third to half the muscle was subsequently divided at its medial border. Mobility was tested continuously; as soon as the muscle part of the flap reached the muscle on the other side of the defect without tension, the dissection was terminated. The flap was sutured in layers with interrupted monofilament resorbable 2/0 sutures. Two drains were placed and kept for 4–6 days, one deep to the muscle and one along the flap in the subcutis.

### Postoperative rehabilitation programmes

Participating centres were obliged to choose one of two prescribed rehabilitation programmes, which had been studied earlier and were considered safe^8,9^.

### Outcomes

The primary endpoint was the proportion of patients underperforming in the timed-stands test (TST) at 6 months after surgery. The TST measures how many seconds it takes to perform 10 stand-up–sit-down sequences^17,18^, and was assessed by a physiotherapist or study nurse. The test metric(s) was compared with age- and sex-matched reference values. The summary measure was the proportion of patients underperforming (that is, exceeding the time limit).

Secondary outcomes were changes from baseline in the TST, maximum step-up height, ability to sit, pain or discomfort, and the use of pain medication at 3, 6, and 12 months after surgery. The maximum step-up height test^19^ was modified to measure height in 5-cm intervals and was performed for both legs. The test metric was maximum step height of an adjustable step that the patient was able to climb on without help. At 3 months, the perineal wound was classified according to the validated^20^ Southampton Wound Assessment Scale^21^, and postoperative complications were classified according to the Clavien–Dindo taxonomy^22^. If present, the following specific complications were also noted: persisting sinus or fistula in the perineal wound; perineal hernia; removal of implant; and excision of a myocutaneous flap in part or whole.

The study protocol^16^ includes quality of life measures as secondary outcomes, which are omitted in the present study.

### Statistical analysis

The sample size was considered in relation to proving non-inferiority of the experimental APCI arm to the control GMF arm in terms of the primary outcome. A power calculation for a binary outcome, using a 5% significance level and 90% statistical power, determined that 38 patients were needed in each arm to demonstrate non-inferiority assuming a non-inferiority threshold of −10% for the difference in underperforming patients (GMF minus APCI). Pretrial estimates of the proportion of patients exhibiting low performance were 40% for the APCI arm and 63% for the GMF arm^23^.

Accounting for attrition and missing data (10%), the goal was to include 85 patients. The primary endpoint was assessed in the modified intention-to-treat (ITT) population and presented as proportions.

Generalized linear models with an identity link and binomial family were used to estimate risk differences for the primary outcome. A one-sided 95% confidence interval (c.i.) was calculated to evaluate the non-inferiority hypothesis, both for unadjusted data and adjusted data for preoperative radiotherapy and TST at baseline to alleviate potential chance confounding. Due to concerns about overparameterization and convergence issues, adjustments were performed one variable at a time. Limited data prevented further adjustments. The same analyses were repeated for the per-protocol population. Missing data for the primary endpoint at 6 months were handled by imputing the 12-month TST measure, if few differences in wound healing occurred between these time points. In addition, multiple chained imputation for the primary outcome was performed as a sensitivity analysis (using treatment, radiotherapy, body mass index, age, test values for the TST at baseline and 3, 6, and 12 months, and maximum step-up height for the right leg at 3, 6, and 12 months). In a post hoc analysis, non-inferiority of the GMF in relation to the APCI (APCI–GMF) was also evaluated.

Comparisons between treatment groups of proportions for nominal variables and mean values for continuous variables were performed using Fisher’s exact test and independent-samples *t* test, respectively. For ordinal variables and skewed continuous data, the Mann–Whitney *U* test was used. All analyses were performed using Stata^®^ version 17 (StataCorp, College Station, TX, USA) and, unless stated otherwise, adhered to the analysis plan in the study protocol^16^.

## Results

### Recruitment

Between 6 September 2011 and the 26 September 2022, 434 patients were assessed for eligibility. Of these, 85 patients with primary rectal cancer fulfilled the eligibility criteria and accepted inclusion. From this group, 44 patients were randomly assigned to an APCI (experimental arm), and 41 patients were randomly assigned to a GMF (control arm). Two patients in the experimental arm were excluded from analysis because they were not operated on or had no reconstruction of the pelvic floor. Thus, 83 patients remained for analysis in the modified ITT population. Of these, seven patients in the experimental arm and eight patients in the control arm were lost to follow-up before the 6-month mark. In the experimental arm, 35 patients received a Permacol™ implant (Sofradim Production facility, Medtronic, Trévoux, France), three patients received a Strattice™ implant (LifeCell Corporation, subsidiary of AbbVie Inc. North Chicago, Illinois, USA), one patient received an unspecified APCI, and three patients were operated on with another reconstruction technique than the assigned one; the latter also occurred for one patient in the control arm (*Fig. 1*). The GMF reconstruction in the control arm was performed by plastic surgeons in five hospitals and by colorectal surgeons in two hospitals (the latter included 50 of 83 patients in the study).

No serious adverse events were reported to the principal investigator (MH).

### Baseline characteristics

*Table 1* lists the demographic and tumour-related variables for the modified ITT population. The mean(standard deviation) age of the entire cohort was 68(10) years and 69% of patients (57 of 83) were male. The American Society of Anesthesiologists fitness grade was I or II in 73% of patients (61 of 83). The median body mass index was 26 (interquartile range (i.q.r.) 23–29) kg/m^2^. Local tumour categories were c (clinical) T3 and cT4 in 36% (30 of 83) and 36% (30 of 83) of patients, respectively, and the median tumour height from the anal verge was 3 (i.q.r. 2–5) cm, measured by rigid sigmoidoscopy. In all, 43% patients (36 of 83) had long-course radiotherapy. The baseline characteristics were mostly evenly distributed across study arms (*Table 1*), whereas the proportion of patients underperforming in the TST tended to be lower in the GMF arm (58%; 21 of 36) than in the APCI arm (71%; 25 of 35).

**Table 1:** Baseline characteristics of 83 patients with primary rectal cancer in the NEAPE study according to study arm

	GMF (*n* = 41)	APCI (*n* = 42)
Age (years), mean(s.d.)	68.5(10.4)	67.7(9.4)
**Sex (*n*)**		
Male	27	30
Female	14	12
**ASA fitness grade (*n*)**		
I	3 of 41	4 of 42
II	27 of 41	27 of 42
III–IV	11 of 41	11 of 42
BMI (kg/m^2^), median (i.q.r.)	25.8 (23.8–28.7)	26.0 (22.4–28.8)
**cT category (*n*)**		
cT1–2	9 of 37	7 of 39
cT3	13 of 37	17 of 39
cT4	15 of 37	15 of 39
**cN category (*n*)**		
cN0	11 of 37	8 of 38
cN1–2	26 of 37	30 of 38
**cM category (*n*)**		
cM0	32 of 37	37 of 38
cM1	5 of 37	1 of 38
**Tumour height from anal verge (cm), median (i.q.r.)**	3.0 (2.0–5.0)	3.0 (2.0–4.0)
**Neoadjuvant radiotherapy (*n*)**		
Short-course 5 × 5 Gy with immediate surgery	11 of 41	14 of 42
Short-course 5 × 5 Gy with delayed surgery	10 of 41	9 of 42
Long-course 25 × 2 Gy	18 of 41	18 of 42
Previous irradiation	2 of 41	1 of 42
**Neoadjuvant chemotherapy (*n*)**		
Yes	19 of 41	21 of 42
No	22 of 41	21 of 42
Patients underperforming in TST (*n*)	21 of 36	25 of 35
**Maximum step-up height (cm), mean(s.d.)**		
Right leg	47.9(12.6)	43.8(11.9)
Left leg	47.6(13.0)	43.7(11.8)
**Minimally invasive surgery (*n*)**		
No	28 of 40	29 of 42
Yes	12 of 40	13 of 42
Operation time (min), median (i.q.r.)	384 (294–423)	355 (281–427)
Intraoperative bleeding (ml), median (i.q.r.)	350 (200–700)	400 (200–600)
**(y)pT category (*n*)**		
0	4 of 33	3 of 39
1	2 of 33	0 of 39
2	5 of 33	11 of 39
3	21 of 33	25 of 39
4	1 of 33	0 of 39
**(y)pN category (*n*)**		
0	23 of 34	19 of 39
1	9 of 34	14 of 39
2	2 of 34	6 of 39
**pM category (*n*)**		
0	29 of 34	37 of 39
1	5 of 34	2 of 39

Numbers for denominators do not sum up to group totals because missing data are not presented in the table. The tumour node metastasis (TNM) staging system subclasses are per the Classification of Malignant Tumours, 8th edition^24^, published by Union for International Cancer Control (UICC). The TST is a test of physical performance. Operation time and intraoperative bleeding volume are rounded to nearest integer. GMF, gluteus maximus myocutaneous flap; APCI, acellular porcine collagen implant; s.d., standard deviation; ASA, American Society of Anesthesiologists; BMI, body mass index; i.q.r., interquartile range; c, clinical; TST, timed-stands test; min, minutes; (y)p, (post-therapy) pathological.

### Operative data

No multivisceral resections except minor vaginal resections were reported for the included patients. Minimally invasive surgery (abdominal part) was performed for 30% of patients (25 of 83). The median operative time was 29 minutes longer in the GMF arm, but this difference was not statistically significant (Mann–Whitney, *P* = 0.827). Intraoperative bleeding was similar in both groups. The postoperative histopathology was uniformly distributed between study arms; most patients had a (y)p (post-therapy) T3 tumour (*Table 1*).

### Primary outcome

**Fig. 2:**
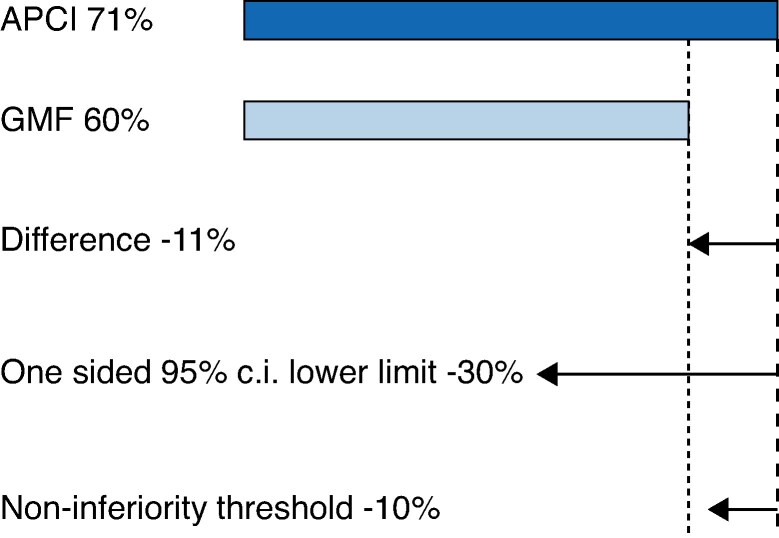
Main results Proportion of patients with low physical performance, defined as underperformance in the timed-stands test 6 months after surgery. Underperformance was measured relative to age- and sex-adjusted reference values. Non-inferiority is achieved when the lower limit of a one-sided 95% c.i. of the difference between groups is higher than the non-inferiority threshold, which was set to −10%. Non-inferiority of the APCI *versus* GMF could not be determined. The GMF group tended to have better physical function, although this was not statistically significant. APCI, acellular porcine collagen implant; GMF, gluteus maximus myocutaneous flap; c.i., confidence interval.

The primary outcome measurement, namely the TST at 6 months, was missing for four patients in the experimental arm and for one patient in the control arm; values for these patients were imputed using their 12-month TST measurements. *Figure 2* illustrates the main findings of this study. Six months after surgery, the percentage of patients who underperformed in the TST was 71% (25 of 35; seven dropouts) in the APCI arm and 60% (20 of 33; eight dropouts) in the GMF arm (*P* = 0.44). In the main modified ITT analysis, the risk difference between groups regarding low performance was −11% (GMF–APCI), with a one-sided 95% c.i. lower limit of −30%. Thus, non-inferiority could not be shown for the APCI because the prespecified non-inferiority threshold of −10% was not met (that is, the lower confidence limit was −30%).

### Sensitivity analysis

After adjustment of the modified ITT analysis with baseline TST values, the 95% one-sided c.i. lower limit was −20%, a level still outside the non-inferiority threshold (*Table 2*).

**Table 2:** Summary of main analysis and sensitivity analyses for non-inferiority of acellular porcine collagen implant *versus* gluteus maximus myocutaneous flap

	TST risk difference at 6 months	
	Observed data only* (*n* = 68)	Multiple imputed data (*n* = 73)
**Main analysis**		
mITT analysis	−11% (−30%, ∞)	−12% (−31%, ∞)
**Sensitivity analyses**		
mITT analysis adjusted for RT	−10% (−29%, ∞)	−11% (−30%, ∞)
mITT analysis adjusted for TST at baseline	−3% (−20%, ∞)	−2% (−19%, ∞)
Per-protocol analysis: unadjusted	−17% (−35%, ∞)	−18% (−36%, ∞)
Per-protocol analysis: adjusted for RT	−18% (−36%, ∞)	−18% (−36%, ∞)
Per-protocol analysis: adjusted for TST at baseline	−4% (−22%, ∞)	−5% (−22%, ∞)

Values in parentheses are one-sided 95% confidence intervals. The TST risk difference is the risk for physical underperformance in the gluteus maximus myocutaneous flap group minus the risk in the acellular porcine collagen implant group. Underperformance was measured relative to age- and sex-adjusted reference values. *TST values missing at 6 months were imputed from the 12-month mark for five patients. TST, timed-stands test; mITT, modified intention-to-treat; RT, radiotherapy.

### Post hoc analysis

In a post hoc analysis, the GMF met the non-inferiority criterion. The difference of underperforming patients was 11% (APCI–GMF) and the lower limit of the one-sided 95% c.i. for that difference was −8% (that is, within the non-inferiority threshold of −10%).

When the study cohort was stratified by radiotherapy modality, no statistically significant differences were observed between the groups in terms of persistent sinus or fistula formation, or in postoperative complications at any time point.

### Secondary outcomes

*Table 3* summarizes the secondary outcomes. There were no substantial differences between study arms in terms of change of TST measurements at 3, 6, or 12 months. For the GMF arm, the maximum step-up height was statistically significantly higher for both legs at 3, 6, and 12 months. There were no important differences between groups regarding ability to sit and pain or discomfort at any time point after surgery. There was a greater use of pain medication in the APCI group at 3 and 6 months after surgery, but this difference was not significant at 12 months. Primary wound healing, measured by the Southampton Wound Assessment Scale at 3 months, was not significantly different between groups, although clear or haemoserous discharge was more common in the APCI than GMF group (10 of 38 *versus* 2 of 32 patients, respectively). Postoperative complications at 3 months did not differ significantly between the two groups.

**Table 3:** Secondary outcomes of 83 patients in the NEAPE study according to study arm

	GMF (*n* = 41)	APCI (*n* = 42)	P*
**TST *versus* baseline measurement (*n*)**			
At 3 months, change from baseline:			0.757
Better	2 of 30	4 of 29	
No change	23 of 30	21 of 29	
Worse	5 of 30	4 of 29	
At 6 months, change from baseline:			0.900
Better	2 of 31	3 of 28	
No change	25 of 31	21 of 28	
Worse	4 of 31	4 of 28	
At 12 months, change from baseline:			0.610
Better	5 of 27	3 of 28	
No change	20 of 27	21 of 28	
Worse	2 of 27	4 of 28	
**Maximum step-up height (cm), mean(s.d.)**			
Right leg, 3 months	44.7(13.8)	37.4(11.6)	0.025†
Right leg, 6 months	45.3(11.8)	39.8(10.0)	0.046†
Right leg, 12 months	48.2(11.7)	38.6(9.1)	0.001†
Left leg, 3 months	45.2(12.8)	37.5(11.8)	0.016†
Left leg, 6 months	45.1(11.7)	39.5(10.3)	0.042†
Left leg, 12 months	47.5(11.3)	38.4(10.2)	0.003†
**Ability to sit at 3 months (*n*)**			0.297
No	8 of 31	14 of 35	
Yes	23 of 31	21 of 35	
**Ability to sit at 6 months (*n*)**			0.752
No	5 of 34	7 of 35	
Yes	29 of 34	28 of 35	
**Ability to sit at 12 months (*n*)**			1.000
No	4 of 31	5 of 33	
Yes	27 of 31	28 of 33	
**Pain/discomfort§, median (i.q.r.)**			
At 3 months	0.0 (0.0–13.5)	0.0 (0.0–20.0)	0.371‡
At 6 months	0.0 (0.0–3.0)	0.0 (0.0–13.0)	0.283‡
At 12 months	0.0 (0.0–10.0)	0.0 (0.0–1.0)	0.792‡
**Change in pain/discomfort, median (i.q.r.)**			
At 3 months	0.0 (0.0–7.0)	0.0 (0.0–18.0)	0.54‡
At 6 months	0.0 (0.0–1.0)	0.0 (0.0–5.0)	0.533‡
At 12 months	0.0 (0.0–10.0)	0.0 (0.0–0.0)	0.651‡
**Use of analgesic tablets at 3 months (*n*)**			0.008
No	26 of 32	22 of 37	
Yes, occasionally	4 of 32	2 of 37	
Yes, daily	2 of 32	13 of 37	
**Use of analgesic tablets at 6 months (*n*)**			0.016
No	28 of 35	30 of 37	
Yes, occasionally	5 of 35	0 of 37	
Yes, daily	2 of 35	7 of 37	
**Use of analgesic tablets at 12 months (*n*)**			1
No	29 of 31	30 of 34	
Yes, occasionally	0 of 31	1 of 34	
Yes, daily	2 of 31	3 of 34	
**SWAS at 3 months (*n*)**			0.289
0: Normal healing	23 of 32	21 of 38	
I: Normal healing with mild bruising or hematoma	2 of 32	3 of 38	
II: Erythema plus other signs of inflammation	2 of 32	2 of 38	
III: Clear or haemoserous discharge	2 of 32	10 of 38	
IV: Pus	2 of 32	1 of 38	
V: Deep or severe wound infection	1 of 32	1 of 38	
**Complications at 3 months (*n*)**			0.917
None	17 of 32	19 of 37	
CD grade I	7 of 32	6 of 37	
CD grade II	6 of 32	8 of 37	
CD grade IIIa	1 of 32	3 of 37	
CD grade IIIb	1 of 32	1 of 37	
**Persistent perineal sinus/fistula at 3 months (*n*)**			0.070
No	24 of 31	19 of 35	
Yes (all)	7 of 31	16 of 35	
Short-course RT, immediate surgery	3 of 10	7 of 11	
Short-course RT, delayed surgery	1 of 7	5 of 8	
Long-course RT, delayed surgery	3 of 13	3 of 15	
Previous RT	0 of 1	1 of 1	
**Persistent perineal sinus/fistula at 6 months (*n*)**			0.383
No	27 of 32	27 of 36	
Yes (all)	5 of 32	9 of 36	
Short-course RT, immediate surgery	4 of 11	5 of 12	
Short-course RT, delayed surgery	0 of 7	2 of 9	
Long-course RT, delayed surgery	1 of 13	2 of 14	
Previous RT	0 of 1	0 of 1	
**Persistent perineal sinus/fistula at 12 months (*n*)**			0.352
No	27 of 28	24 of 28	
Yes (all)	1 of 28	4 of 28	
Short-course RT, immediate surgery	1 of 9	1 of 10	
Short-course RT, delayed surgery	0 of 5	1 of 8	
Long-course RT, delayed surgery	0 of 13	2 of 9	
Previous RT	0 of 1	0 of 1	
**Perineal hernia at 12 months (*n*)**			1.000
No	26 of 27	25 of 25	
Yes	1 of 27	0 of 25	

Note, numbers do not add up to group totals because of missing data. §Pain/discomfort was measured using a visual analogue scale. GMF, gluteus maximus myocutaneous flap; APCI, acellular porcine collagen implant; TST, timed-stands test; s.d., standard deviation; i.q.r., interquartile range; SWAS, Southampton Wound Assessment Scale; CD, Clavien–Dindo; RT, radiotherapy. *Fisher’s exact test, except †Students *t* test and ‡Mann–Whitney *U* test.

A persistent perineal fistula or sinus was more prevalent in the APCI than GMF group at all timepoints, but this difference decreased over time and was not statistically significant. No APCI was removed during follow-up and none of the GMFs was resected.

## Discussion

In this multicentre Nordic trial, APCI did not demonstrate non-inferiority to GMF regarding physical impairment. This result was robust to sensitivity analyses, and the risk difference was in favour of the GMF group. Although not prespecified, a post hoc analysis suggested non-inferiority of GMF relative to APCI. Moreover, the maximum step-up height test favoured the GMF group at all time points after surgery. There were no important differences in other secondary outcomes, although there were indications that a persistent fistula or sinus was slightly more common and the daily use of pain medication 3 months after surgery was more prevalent in the experimental group.

The strengths of this study include its randomized design and multicentre setting, enhancing internal and external validity, respectively. The primary outcome is patient oriented, aimed at measuring physical impairment, perceived as one of the main disadvantages of using a GMF.

NEAPE is the second randomized study of reconstruction options in which all included patients have undergone a full extended APE procedure, and the first to evaluate a biological mesh *versus* a myocutaneous flap. In addition, this is the first study to evaluate and compare physical function in terms of mobility, ability to sit, and muscle strength in the lower extremities after extended APE procedures. Another high-quality randomized clinical trial (BIOPEX) conducted by Blok *et al*.^6^ randomized extended APE patients to primary closure or with biological mesh, which yielded similar results in terms of wound healing but a long-term reduced perineal hernia rate in the mesh group. The follow-up BIOPEX-2 trial by Kreisel *et al*.^12^ allocated APE patients to either primary closure or a muscle-sparing gluteal turnover flap, which is a less invasive alternative to the GMF because only skin and subcutaneous fat is involved; the extent of the extralevator APE was tailored to the oncological requirements. In that study, the primary outcome (wound complications measured by the Southampton Wound Assessment Scale)was similar between study arms, although the share of presacral abscesses was lower in the flap group^12^. Comparisons to the present study are difficult for a number of reasons, not the least because the patient population consists of extended APE only in NEAPE, whereas BIOPEX-2 included conventional APE as well as extended APE. In addition, the BIOPEX trials did not study the GMF, whereas NEAPE did not evaluate primary closure without reconstruction.

Although the use of a GMF should, in theory, result in difficulties in sitting, walking, and standing up, there were no significant differences in these parameters between groups. In fact, there was a counterintuitive trend towards better function in the GMF group. Baseline imbalance in physical performance may have influenced the primary outcome despite randomization. Even though the same randomization process successfully balanced other preoperative measures across groups, it is recognized that this imbalance may influence the interpretation of the results. Therefore, sensitivity analyses were undertaken to account for its potential impact. The adjustment for such differences did render the study arms more comparable; however, non-inferiority was still not evident.

In addition to the reconstruction methods mentioned above, several other techniques using different tissue flaps have been described, such as gluteal V–Y advancement flaps^25^, inferior gluteal artery perforator flaps^26^, gluteal fold flaps^25^, vertical or oblique rectus abdominis myocutaneous flaps^27^, gracilis flaps^28^, and internal pudendal artery perforator flaps^29^. Unfortunately, no randomized studies comparing these techniques exist, and most cohort studies are retrospective, which limits the value of comparisons to the present study.

This study has limitations. Blinding was not feasible. Although blinding is admittedly difficult to accomplish in surgical trials, non-blinding may have resulted in detection bias. The present trial faced numerous difficulties during the recruitment period, which was longer than anticipated. Some centres faced logistical difficulties, such as unexpected lack of capacity for prolonged surgery, including reconstructions with plastic surgeon expertise or dedicated physiotherapist services, a situation that made further randomization impossible. Finally, the sample size, although conforming to the derived power calculation, was still too low to permit subgroup analyses. There was a higher number of dropouts (17.6%) than anticipated (10%) in the original power calculation, which had been performed to achieve 90% power. This substantial proportion of dropouts inevitably reduced the statistical power of the study. However, the power remained well above 80% with 68 patients analysed, because achieving 80% power with the same effect size and margin would require 54 patients.

NEAPE did not demonstrate non-inferiority of APCI compared with GMF for perineal reconstruction after extended APE. The direction of effect favoured the flap reconstruction, suggesting the possibility of worse functional outcomes with the implant.

## Supplementary Material

zrag079_Supplementary_Data

## Data Availability

Anonymized patient data can be provided to individual researchers upon request if they consent to publish group-level data only. The study’s ethics approval and the European GDPR regulation prohibit data publication at the individual level.
