# Supplementary material for: Collagen implant versus gluteus maximus flap for perineal closure after extended abdominoperineal excision: NEAPE randomized clinical trial
Source: BJS Open. 2026 Jun 24;10(3):zrag079. doi: 10.1093/bjsopen/zrag079 (PMC13293247; doi:10.1093/bjsopen/zrag079)
Supplement: zrag079_Supplementary_Data [file zrag079_supplementary_data.zip › Rutegård_2024_NEAPE abstr BJS.pdf]

Abstract citation ID: znae175.060

## 71698 - A multicenter randomized trial: Collagen implant vs myocutaneous flap for reconstruction after extralevator abdominoperineal excision of the rectum

Martin Rutegård[1,2], Jörgen Rutegård[1], **Markku M Haapamäki**[1]
[1]*Department of Diagnostics and Intervention, Surgery, Umeå university, Umeå, Sweden*
[2]*Wallenberg Centre for Molecular Medicine, Umeå University, Umeå, Sweden*

**Introduction:** The procedures to address the defect in the lesser pelvis following an ‘extralevator’ abdominoperineal excision (ELAPE) for rectal cancer have been explored only sparsely. The objective was to determine whether a porcine-collagen implant or a gluteus maximus myocutaneous flap for reconstruction is superior.

**Method:** This study was a multicenter, randomized clinical trial (the NEAPE study). Eligible participants were rectal cancer patients undergoing ELAPE, where the remaining muscles could not be closed in the midline, necessitating adjuncts for closure. Randomization was computer-generated, concealed, and stratified by hospital and preoperative radiotherapy regimen. The experimental arm consisted of a porcine-collagen implant, whilst the control arm comprised a gluteal maximus myocutaneous flap. The primary outcome was physical performance six months after surgery, measured using the timed-stands test (TST). Secondary outcomes included perineal wound healing and sitting ability.

**Result:** In total, 83 patients (57 men and 26 women) from 8 hospitals were included. The mean age was 68 years (range: 41–93). Some 78 patients had preoperative radiotherapy, 40 had preoperative chemotherapy and 38 had a preoperative combination of both.

The proportion of patients within reference values of TST matched for age and sex was 28.6 per cent for the collagen implant group and 39.4 per cent for the myocutaneous flap group (missing excluded), p=0.44. Results of primary and two secondary outcomes are shown in Table 1.

**Discussion:** The NEAPE study was unable to show superiority for any of the two reconstructive techniques, which seem equally effective regarding postoperative physical function, wound complications and ability to sit.

**Table 1.** Randomized NEAPE study outcomes 6 months post-operatively with comparison of reconstruction of pelvic floor with a porcine collagen implant or gluteus maximus myocutaneous flap.

| *Outcome* | *Porcine-collagen implant* | *Gluteus Maximus flap* | *Total number* | *P-value* |
|---|---|---|---|---|
| Timed-stands test within reference values matched for age and sex | | | | |
| Yes | 10 | 13 | 23 | 0.44* |
| No | 25 | 20 | 45 | |
| Number of observations | 35 | 33 | 68 | |
| Missing observations | 7 | 8 | 15 | |
| Total number | 42 | 41 | 83 | |
| Clearly disturbed wound healing with Southampton Wound Assessment Scale grades III, IV or V merged | | | | |
| Yes | 8 | 5 | 13 | 0.55* |
| No | 29 | 28 | 57 | |
| Number of observations | 37 | 33 | 70 | |
| Missing observations | 5 | 8 | 13 | |
| Total number | 42 | 41 | 83 | |
| Ability to sit comfortably in an unchanged position for 10 minutes | | | | |
| Yes | 28 | 29 | 57 | 0.75* |
| No | 7 | 5 | 12 | |
| Number of observations | 35 | 34 | 69 | |
| Missing observations | 7 | 7 | 14 | |
| Total number | 42 | 41 | 83 | |

*Fisher’s exact test calculated without missing observations.

Downloaded from https://academic.oup.com/bjs/article/111/Supplement_7/znae175.060/7726053 by guest on 22 October 2025
