# Supplementary material for: Collagen implant versus gluteus maximus flap for perineal closure after extended abdominoperineal excision: NEAPE randomized clinical trial
Source: BJS Open. 2026 Jun 24;10(3):zrag079. doi: 10.1093/bjsopen/zrag079 (PMC13293247; doi:10.1093/bjsopen/zrag079)
Supplement: zrag079_Supplementary_Data [file zrag079_supplementary_data.zip › Supplementary_Material.docx]

**Collagen implant versus gluteus maximus flap for perineal closure after extended abdominoperineal excision: NEAPE randomized clinical trial**

**Authors:** Martin Rutegård, M.D., Ph.D. (1), Johan Svensson, Ph.D. (1,2), Jörgen Rutegård, M.D., Ph.D. F.R.C.S. (1), Tero Rautio, M.D., Ph.D. (3), Per J Nilsson, M.D., Ph.D. (4), Olle Sjöström, M.D., Ph.D. (1), Marie-Louise Lydrup, M.D., Ph.D. (5), Christoffer Odensten, M.D., Ph.D. (1), Andreas Söderström, M.D. (1), Michael Dahlberg, M.D., Ph.D. (1), Markku M Haapamäki, M.D., Ph.D. (1)

### **Affiliations:**

1) Department of Diagnostics and Intervention, Surgery, Umeå University, Umeå, Sweden.

2) Department of Statistics, Umeå School of Business, Economics and Statistics, Umeå University, Umeå, Sweden.

3) Oulu University Hospital, Medical Research Centre Oulu, Oulu, Finland

4) Department of Molecular Medicine and Surgery, Karolinska Institutet, Stockholm, Sweden.

5) Department of Surgery, Skåne University Hospital, Malmö, Lund University, Lund, Sweden.

**Corresponding author.**

Markku M Haapamäki

Department of Diagnostics and Intervention, Umeå University

SE-90185 Umeå, Sweden

Email: [markku.haapamaki@umu.se](mailto:markku.haapamaki@umu.se)

Telephone: +46-90-785 1153

Mobile phone: +46-73-063 8553

Fax: +46-90-785 1156

**ORCID ID**; 0000-0001-6172-8685

**Twitter**

**Supplementary Materials - Index**

| **Supplementary Methods** |  |
| --- | --- |
|  | *page 2* |
| *Size of the randomization blocks* | *page 2* |
| **Supplementary Results** |  |
|  | *page 3* |
| *The number of operated patients per hospital* | *page 3* |
|  |  |
| **Consort checklist** | *page 4* |
|  |  |
| **Study protocol** | *page 8* |
|  |  |
|  |  |

**Supplementary Methods**

*The info on the size of the randomization blocks was concealed during the inclusion phase of the study but can now be disclosed. The block size was either 4 or 6 and came in a random order.*

**Supplementary Results**

*The number of operated patients per hospital was Karolinska 11, Malmö 13, Oulu, Finland 5, Sunderby 30, Umeå 20, Uppsala 3, Västerås 1. Östersund regional hospital organized follow-up for 7 patients from the Östersund region that underwent surgery in Umeå.*

|  | Section/topic | No | CONSORT 2025 checklist item description | Reported on page no. |
| --- | --- | --- | --- | --- |
|  | **Title and abstract** | | |  |
|  | Title and structured abstract | 1a | Identification as a randomised trial | 1 |
|  |  | 1b | Structured summary of the trial design, methods, results, and conclusions | 3-4 |
|  | **Open science** | | |  |
|  | Trial registration | 2 | Name of trial registry, identifying number (with URL) and date of registration | 4 |
|  | Protocol and statistical analysis plan | 3 | Where the trial protocol and statistical analysis plan can be accessed | 4 |
|  | Data sharing | 4 | Where and how the individual de-identified participant data (including data dictionary), statistical code and any other materials can be accessed | 2 |
|  | Funding and conflicts of interest | 5a | Sources of funding and other support (eg, supply of drugs), and role of funders in the design, conduct, analysis and reporting of the trial | 4 |
|  |  | 5b | Financial and other conflicts of interest of the manuscript authors | 2 |
|  | **Introduction** | | |  |
|  | Background and rationale | 6 | Scientific background and rationale | 5 |
|  | Objectives | 7 | Specific objectives related to benefits and harms | 5, 8 |
|  | **Methods** | | |  |
|  | Patient and public involvement | 8 | Details of patient or public involvement in the design, conduct and reporting of the trial | N/A |
|  | Trial design | 9 | Description of trial design including type of trial (eg, parallel group, crossover), allocation ratio, and framework (eg, superiority, equivalence, non-inferiority, exploratory) | 6 |
|  | Changes to trial protocol | 10 | Important changes to the trial after it commenced including any outcomes or analyses that were not prespecified, with reason | 10 |
|  | Trial setting | 11 | Settings (eg, community, hospital) and locations (eg, countries, sites) where the trial was conducted | 3 and 6 |
|  | Eligibility criteria | 12a | Eligibility criteria for participants | 6 |
|  |  | 12b | If applicable, eligibility criteria for sites and for individuals delivering the interventions (eg, surgeons, physiotherapists) | Study protocol |
|  | Intervention and comparator | 13 | Intervention and comparator with sufficient details to allow replication. If relevant, where additional materials describing the intervention and comparator (eg, intervention manual) can be accessed | 7-8 |
|  | Outcomes | 14 | Prespecified primary and secondary outcomes, including the specific measurement variable (eg, systolic blood pressure), analysis metric (eg, change from baseline, final value, time to event), method of aggregation (eg, median, proportion), and time point for each outcome | 8-9 |
|  | Harms | 15 | How harms were defined and assessed (eg, systematically, non-systematically) | 8 |
|  | Sample size | 16a | How sample size was determined, including all assumptions supporting the sample size calculation | 9 |
|  |  | 16b | Explanation of any interim analyses and stopping guidelines | Study protocol |
|  | Randomisation: |  |  |  |
|  | Sequence generation | 17a | Who generated the random allocation sequence and the method used | 7 |
|  |  | 17b | Type of randomisation and details of any restriction (eg, stratification, blocking and block size) | 6-7 |
|  |  |  |  | **Reported on page no.** |
|  | Allocation concealment mechanism | 18 | Mechanism used to implement the random allocation sequence (eg, central computer/telephone; sequentially numbered, opaque, sealed containers), describing any steps to conceal the sequence until interventions were assigned | 6-7 |
|  | Implementation | 19 | Whether the personnel who enrolled and those who assigned participants to the interventions had access to the random allocation sequence | 6-7 |
|  | Blinding | 20a | Who was blinded after assignment to interventions (eg, participants, care providers, outcome assessors, data analysts) | 7 |
|  |  | 20b | If blinded, how blinding was achieved and description of the similarity of interventions | N/A |
|  | Statistical methods | 21a | Statistical methods used to compare groups for primary and secondary outcomes, including harms | 9-10 |
|  |  | 21b | Definition of who is included in each analysis (eg, all randomised participants), and in which group | 11 |
|  |  | 21c | How missing data were handled in the analysis | 10 |
|  |  | 21d | Methods for any additional analyses (eg, subgroup and sensitivity analyses), distinguishing prespecified from post hoc | 10 |
|  | **Results** | | |  |
|  | Participant flow, including flow diagram | 22a | For each group, the numbers of participants who were randomly assigned, received intended intervention, and were analysed for the primary outcome | 11 |
|  |  | 22b | For each group, losses and exclusions after randomisation, together with reasons | 10-11 |
|  | Recruitment | 23a | Dates defining the periods of recruitment and follow-up for outcomes of benefits and harms | 10 |
|  |  | 23b | If relevant, why the trial ended or was stopped | N/A |
|  | Intervention and comparator delivery | 24a | Intervention and comparator as they were actually administered (eg, where appropriate, who delivered the intervention/comparator, how participants adhered, whether they were delivered as intended (fidelity)) | 11 |
|  |  | 24b | Concomitant care received during the trial for each group | 11 |
|  | Baseline data | 25 | A table showing baseline demographic and clinical characteristics for each group | Table 1 |
|  | Numbers analysed,  outcomes and estimation | 26 | For each primary and secondary outcome, by group:  ● the number of participants included in the analysis  ● the number of participants with available data at the outcome time point  ● result for each group, and the estimated effect size and its precision (such as 95% confidence interval)  ● for binary outcomes, presentation of both absolute and relative effect size | Tables 2-3 |
|  | Harms | 27 | All harms or unintended events in each group | Table 3 |
|  | Ancillary analyses | 28 | Any other analyses performed, including subgroup and sensitivity analyses, distinguishing pre-specified from post hoc | 13 |
|  | **Discussion** | | |  |
|  | Interpretation | 29 | Interpretation consistent with results, balancing benefits and harms, and considering other relevant evidence | 14-15 |
|  | Limitations | 30 | Trial limitations, addressing sources of potential bias, imprecision, generalisability, and, if relevant, multiplicity of analyses | 17 |

Citation: Hopewell S, Chan AW, Collins GS, Hróbjartsson A, Moher D, Schulz KF, et al. CONSORT 2025 Statement: updated guideline for reporting randomised trials. BMJ. 2025; 388:e081123. <https://dx.doi.org/10.1136/bmj-2024-081123>
© 2025 Hopewell et al. This is an Open Access article distributed under the terms of the Creative Commons Attribution License (<https://creativecommons.org/licenses/by/4.0/>), which permits unrestricted use, distribution, and reproduction in any medium, provided the original work is properly cited.

*We strongly recommend reading this statement in conjunction with the CONSORT 2025 Explanation and Elaboration and/or the CONSORT 2025 Expanded Checklist for important clarifications on all the items. We also recommend reading relevant CONSORT extensions. See [www.consort-spirit.org](http://www.consort-spirit.org).

**Confidential information**

The information contained in this protocol is confidential and is intended for the use of clinical investigators. It is the property of the Department of Surgery at Umeå University and should not be copied or distributed to persons not involved in the process of evaluating the study.

Protocol

for the

Nordic Extended Abdominoperineal Excision study

(NEAPE)

Acellular porcine collagen implant (APCI) versus gluteus maximus myocutaneous flap (GMF) for reconstruction of pelvic floor after extended abdominoperineal excision of rectum (EAPE)

APCI v GMF

Protocol identifier: NEAPE-2010-335-31M

Comprehensive Version 16, 2012-09-13

Markku Haapamäki, Ph.D., M.D.

Jörgen Rutegård, Ph.D., M.D.

Department of Surgical and Perioperative Sciences

Umeå University

**Table of contents**

**Affiliations:** 1

Introduction 10

Objectives 11

Primary objective 11

Secondary objectives 11

Investigational plan 11

Endpoints of study 11

Hypotheses of study 12

Summary of study design 12

Flowchart of NEAPE study 14

Investigator information 16

• Final report signature 17

Study population 17

• Entry procedures 17

Criteria for enrolment 17

Eligibility for enrolment 19

• Disease diagnostic criteria 20

• Patient assignment 20

Discontinuations 20

Surgical methods 21

• Preoperative measures 21

• First part of the operation 21

• Reconstruction of pelvic floor with acellular porcine collagen implant (APCI) 22

• Reconstruction of pelvic floor with gluteus maximus myocutaneous unilateral flap (GMF) 22

22

Compliance 23

Blinding 23

Concomitant therapy 23

Postoperative rehabilitation programmes 23

Evaluation of outcome measures and safety 23

• Measures of physical performance 23

• Measures of complications including the surgical and oncologic result 24

• Safety measures, early postoperative complications, and other clinical adverse events 25

Quality control and quality assurance 26

Data analysis methods 26

Sample size 26

Interpretation of noninferiority 26

Data to be analyzed 27

• Patient disposition 27

• Patient characteristics 27

• Primary analysis 27

• Secondary analyses 27

• Subgroup analyses 28

• Safety analyses 28

• Interim analyses 28

Informed consent, ethical review and regulatory considerations 29

Informed consent 29

Ethical review 29

Regulatory considerations 29

References 30

Appendix 32

• **Form S1:** **Surgeon, first postoperative visit, workup and TNM** 32

• **Form S2**: **Surgeon, early complications within 30 d** 32

• **Form S3: Surgeon, all postoperative visits** 32

• **Form S4: Surgeon, late complications, postoperative visits later than 30 days** 32

• **Form P1: Physiotherapist, pre-treatment** 32

• **Form P2: Physiotherapist, test form** 32

Form P3: Physiotherapist or study nurse, postoperative 33

NEAPE uppgifter om RT och OP 33

Informerat samtycke 36

Protocol signature 37

Amendment History / Change Log 38

Description of changes 38

Rationale for amendment 39

# Introduction

Abdominoperineal excision (APE) of the rectum is the potentially curative operation for rectal carcinomas too low for reconstructive surgery, especially if the levator and sphincter musculature is infiltrated[1]. Preoperative chemo radiotherapy is often used.

The local recurrence rate after APE has been reported to be from 5% up to 47% [2-4]. Consequently, much interest has focused on improved surgical technique, in which the levator musculature is included in the excision by division laterally close to the pelvic wall, creating a cylindrical specimen, as described already by Miles[1, 5, 6]. Encouraging oncological results have been reported [2, 7]^.^ This enlarged operation, when the levator musculature is excised en bloc with the rectum, creates a large defect. Primary closure is often not possible, and reconstruction with prosthetic material or a myocutaneous flap is necessary to avoid a perineal hernia. Implantation of a collagen sheet has shown preliminary good results[8].

Reconstruction with a gluteus maximus myocutaneous flap has the advantages to a trans-abdominal flap of not damaging the abdominal wall, as well as not implanting foreign material. Therefore we have proposed that this study should only use a gluteus maximus myocutaneous flap. Promising results with low local complication rate have recently been reported [7]. However, the gluteus maximus muscle is a strong hip extensor and important for posture and balance. As far as we know, the functional outcome and quality of life (QoL) after this method of reconstruction have not been compared to alternative techniques earlier.

The current study is by definition a comparative effectiveness research project.

# Objectives

## Primary objective

To show if EAPE can be performed with APCI resulting in better outcome in physical performance compared to reconstruction with unilateral GMF (current standard).

To show superiority or noninferiority of test operation (APCI).

## Secondary objectives

- 1. To study which of the techniques result in:
     - 1. better primary wound healing
       2. less late complications including inability to sit, pain and discomfort in gluteal region
       3. greater improvement or lesser impairment of quality of life after reconstruction of floor of lesser pelvis
  2. To study if the different rehabilitation programmes used reflect differences in outcome measures in subgroup analyzes for both methods of reconstruction
  3. To compare the health economic costs with the two techniques and to perform a cost-utility analysis (QALYs gained). The health economic analysis is optional to participating centres. Costs and health economics will be calculated only in centres that have routine registration (in electronic databases) of operation time, time at ICU, days in hospital, connected reoperations, connected readmissions and outpatient visits related to the index operation.

# Investigational plan

## Endpoints of study

1. Primary endpoint: Performance in Timed-stands test (TST)[9, 10]reflecting physical performance at 6 months from operation.
2. Secondary endpoints:
   1. Change in physical performance (3 months, 6 months and 1 year compared to preoperative)
   2. Primary wound healing assessed with the Southampton Wound Assesment Scale (SWAS)[11] at 3 months from operation
   3. Complications according to classification by Dindo-Clavien[12] (3 months, 6 months and 1 year)
   4. Proportion of persistent perineal sinus or fistula (3 months, 6 months, 1 year)
   5. Ability to sit (3 months, 6 months, 1 year)
   6. Change of pain and discomfort in gluteal region measured with VAS (3 months, 6 months and 1 year compared to preoperative)
   7. Change of quality of life measured with EQ-5D and EORTC forms C30 and CR29 (3 months, 6 months and 1 year compared to preoperative)
   8. Quality of life spot measures at 3 months, 6 months and 1 year
   9. Length of hospital stay, costs of surgical treatments and QALYs gained at one year
   10. Local recurrence during study period

## Hypotheses of study

#### Primary hypothesis:

1. The reconstruction of lesser pelvis floor with APCI will result in better physical performance in operated patients compared to patients operated on with GMF.

#### Secondary hypotheses:

1. The assumed decrease of physical performance after the APCI operation will be less than after the GMF operation.
2. The reconstruction of lesser pelvis floor with APCI will result in better wound healing and less wound complications than the GMF operation.
3. The reconstruction of lesser pelvis floor with APCI will result in better ability to sit and lesser pain and discomfort in the gluteal region than the GMF operation,
4. The reconstruction of lesser pelvis floor with APCI will result in better quality of life than the GMF operation.
5. The reconstruction of lesser pelvis floor with APCI will result in lower costs of treatment and more QALYs gained than the GMF operation.
6. The local recurrence rate is not related to the method of reconstruction of lesser pelvis

#### Noninferiority hypothesis:

1. The reconstruction of lesser pelvis floor with APCI will result in clinically equivalent physical performance provided that results will be within defined noninferiority margins, see below.

#### Noninferiority margin:

The noninferiority margin is set to 10%. Interpretation, see section Data analysis methods.

## Summary of study design

The study is multi-centre, prospective randomised and with parallel assignment. Centres that treat locally advanced rectal cancers with the extended abdominoperineal excision of rectum (EAPE)[7] can participate provided that:

1. the operative technique is standardized according to this protocol, see section Surgical methods
2. the centre/unit has resources for examinations of participants by a physiotherapist or a nurse
3. the centre/unit has one investigator in charge of the study locally
4. the centre/unit has an operative volume that enables at least 6 patients to be included/randomised during the anticipated three year study phase for inclusions

Centres that do not operate the rectal cancers included in this study can participate by arranging the preoperative examination and physical tests as well as follow-up of patients that are referred to other centres for the operation. In these cases the operating centre cares for the randomisation, operation and start of postoperative rehabilitation while the study follow-up and final rehabilitation can be completed at the patients’ primary referral hospital. The primary referral hospital needs a site investigator in charge of study patients just like centres that do the operations.

Patients with primary or recurrent cancers of rectal origin can be included but individual patients can be included only once. Concomitant therapies are allowed and preoperative or postoperative radiation therapy and/or chemotherapy may be given or not according to local multidisciplinary team (MDT) decisions.

The participants are examined by a physiotherapist or a study nurse who is familiar with the protocol. Examinations of physical performance, wound healing and registration of quality of life and background information takes place:

1. within one week before the start of radiotherapy (or operation if examination before RT can not be accomplished) (wound healing excluded)
2. three months after the operation
3. six months after the operation
4. one year after the operation
5. at one late time point 2-5 years after the operation if previous analyses indicates a need for long time follow-up. The long time follow-up is optional for participating centres and will be decided later.

A separate detailed protocol of standardized examination procedures will be available for the physiotherapists and nurses involved in the study. A pre-study meeting for standardization of examinations will be arranged for participating centres.

The standardisation of operations will be secured with site visits from (or to) Stockholm Karolinska Sjukhuset Solna where the standard for GMF and APCI operations is defined.

The enrolment of a patient in the study is finalized with the randomization procedure on Internet. The web application at [www.norrlandskirurgi.se](http://www.norrlandskirurgi.se/) is used for randomization.

The randomization is computed in blocks and stratified by:

- Centre
- Mode of preoperative radiotherapy (RT)
  - Short preoperative RT, (5 x 5 GY), **operation within one week from last RT dose**
  - Short preoperative RT, (5 x 5 GY) or long preoperative RT (25 x 1,8/2 GY), **operation 4 - 8 weeks from last RT dose**
  - Earlier RT for organs in the lesser pelvis in connection to a previous therapy but no additional RT before the current operation or other RT dose or timing of operation

The stratification and block randomization ensures that all above defined subgroups of patients will contain patients allocated evenly (or close to evenly) to both study arms. The size of the randomisation blocks will vary within predefined limits and the size of the block under use will be chosen by random from the alternatives given of limits.

### Flowchart of NEAPE study

*Surgeon and study nurse*

MDT conference

- Identify APE and EAPE candidate patients =eligible rectal cancer patients
- Study nurse enter identified patients in register at www.norrlandskirurgi.se

*Study nurse*

For EAPE candidates, send invitation for preoperative visit to surgeon and physiotherapist or study nurse

- Inform physiotherapist and book the visit
- Include patient information of NEAPE study with invitation to patient
- Include preoperative quality of life forms
- Include information of routine registration in study register and CRC register

*Surgeon*

Operation

- Check for eligibility
- Operate according to allocation and NEAPE study standard
- Randomise/Enrol if decision of standard EAPE can be made only at this point
- Follow NEAPE perioperative standards

*Surgeon*

Preoperative visit to attending surgeon

- Check for eligibility
- Inform patient of NEAPE study
- Get informed consent if conditions met and file the document
- Randomise/Enrol if decision of standard EAPE can be made

*Physiotherapist or study nurse*

Preoperative visit to physiotherapist (before RT if possible)

- Fill preoperative form (P1)
- Check and collect QoL forms and inform patient if necessary for complete data
- Perform physical tests and register results on form (P2)
- Register all collected data into the electronic database on Internet

Follow-up

*Study nurse* Weekly check-up that all patients operated on with APE or EAPE at the hospital are either randomised or registered and that correct/completed registration data is saved

- This will be the final data for non-randomised patients

*Study nurse* Send invitation for postoperative visit at 30 days

- Include postoperative quality of life forms

*Surgeon*

**30 days** postoperative NEAPE follow-up visit to attending surgeon

- Fill form of staging and TNM classification (form S1)
- Check for early complications and register on form (S2)
- Classify wound healing and all complications on form (S3)

*Study nurse* Send invitation for postoperative visit at 3 months

- Include postoperative quality of life forms

*Physiotherapist or study nurse*

**3 months** postoperative NEAPE follow-up visit to physiotherapist

- Check and collect QoL forms and inform patient if necessary for complete data
- Perform physical tests and register results on form (P2)
- Fill postoperative form (P3)
- Register all collected data into the electronic database on Internet

*Surgeon*

**3 months** postoperative NEAPE follow-up visit to attending surgeon

- Check for late complications and register on form (S4)
- Classify wound healing and all complications on form (S3)

*Study nurse* Send invitation for postoperative visit at 6 months

- Include postoperative quality of life forms

*Physiotherapist or study nurse*

**6 months** postoperative NEAPE follow-up visit to physiotherapist

- Check and collect QoL forms and inform patient if necessary for complete data
- Perform physical tests and register results on form (P2)
- Register all collected data into the electronic database on Internet

*Surgeon*

**6 months** postoperative NEAPE follow-up visit to attending surgeon

- Check for late complications and register on form (S4)
- Classify wound healing and all complications on form (S3)

*Study nurse* Send invitation for postoperative visit at 12 months

- Include postoperative quality of life forms

## Investigator information

*Physiotherapist or study nurse*

**12 months** postoperative NEAPE follow-up visit to physiotherapist

- Check and collect QoL forms and inform patient if necessary for complete data
- Perform physical tests and register results on form (P2)
- Register all collected data into the electronic database on Internet

*Surgeon*

**12 months** postoperative NEAPE follow-up visit to attending surgeon

- Check for late complications and register on form (S4)
- Classify wound healing and all complications on form (S3)

Investigators in this multicenter study will be identified and approved by the primary investigator (PI) and the central study administration at the Department of Surgery, Umeå University. We aim to identify one investigator from each participating centre and the investigators are specified in the registration of the study at www.clinicaltrials.gov.

### Final report signature

The PI at the Department of Surgery, Umeå University (PI of the central study administration) will sign the final clinical study report for this study, confirming that to the best of his knowledge, the report accurately describes the conduct and results of this study.

We aim to recruit co-authors for the reports of this study among the investigators in participating centres. The investigators in three centres with the highest proportion of enrolments from patients undergoing an EAPE operation in this study will be offered co-authorship for reports and all other investigators will be acknowledged as participating investigators. If five or less centres are involved, one site investigator from each centre will be offered co-authorship.

## Study population

### Entry procedures

Patients with primary or secondary malignancies of the rectum are screened for enrolment during workup for assessing pre-treatment stage of the disease. The screening process has not to be documented and the accomplishment may vary between participating centres.

### Criteria for enrolment

##### Definitions

For this study, the following definitions are used:

**Enter** The act of assessing eligibility of a patient for the study. All patients that are planned for APE or EAPE should be entered (registration). The registration process can be performed before enrolment/randomization (recommended) or at the same time automatically when doing enrolment/randomization (or postoperatively for those not randomised/enrolled).

**Enrol** The act of assigning a patient to a treatment alternative by randomization in the study. When the randomization is done on the web application the patient is registered with status entered and enrolled. The informed consent document must be signed by the patient before enrolment.

**Register entry without randomization**

*If the patient cannot be enrolled (randomised) because of intraoperative or preoperative circumstances the entry must be registered via the web application without randomization*. Choose “registration of entry with no randomization”. *All patients that undergo APE or EAPE in participating centres during the inclusion phase of the study must be registered* via the web application where information of eligibility is registered.

**Registered patients**

All patients registered (entered and enrolled or only entered) are those who are assessed for eligibility when they are planned for APE or EAPE during the inclusion phase of the study.

##### Recommended procedure

We recommend that a study nurse enter/register a patient immediately after a MDT conference were a decision of either APE or EAPE is made. We also recommend that the patient should be informed early, in conjunction with the invitation to a preoperative visit, about being included in the study register and national colorectal cancer register during the process.

Later when the surgeon examines the patient preoperatively, the patient can be informed about the study, asked about participation, informed consent can be obtained, and the patient can be enrolled/randomised if an EAPE operation is decided and criteria fits. The randomization can be postponed to the operating room (intra-operative randomization) if there is uncertainty of fulfilment of inclusion criteria up till this point.

If the patient refuses participation and do not want registration or enrolment and the registration was already done by the nurse, a request of erasing the registration should be communicated with the study administration. This applies only for patients that do not want to be registered. Note that patients can refuse enrolment in the study but still accept registration that simply means that the study administration knows about that an APE or EAPE operation is planned or executed. In these cases the registration stays but the patient is not randomised/enrolled.

We recommend that during the inclusion phase of the study, the study nurse do a weekly check-up for patients operated during the past week with APE or EAPE. The nurse should secure that all these patients are either randomised or registered with limited but correct data on the study homepage at [www.norrlandskirurgi.se](http://www.norrlandskirurgi.se). The data for nonrandomised patients will be final at this stage and the data is later necessary for the CONSORT diagram of final reports.

**Figure 1. Illustration of study design and patient flow to operation.**


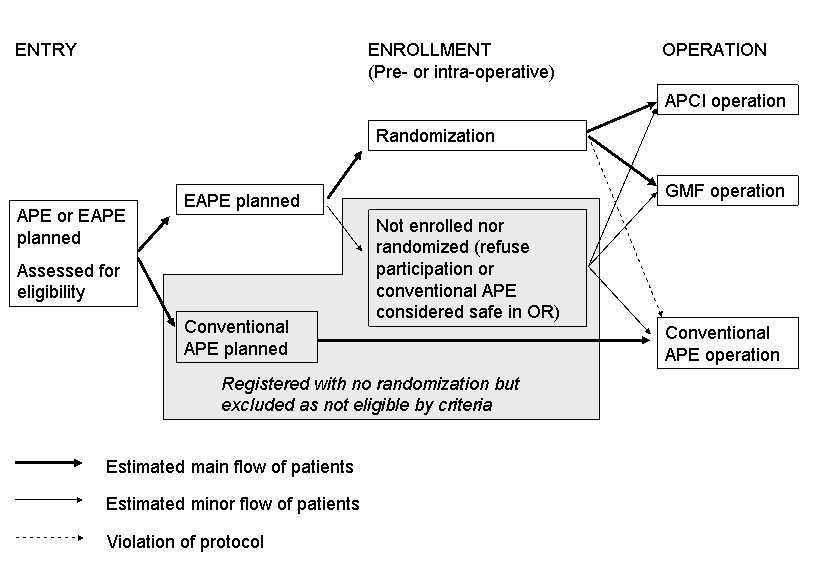


### Eligibility for enrolment

##### Inclusion criteria

Adult (18 years or older) rectal cancer patients where **wide resection** of pelvic floor muscles together with rectum and anal canal **have made reconstruction of pelvic floor necessary** i.e. primary suture of pelvic floor in the midline is not possible. Resection of coccyx is optional.

##### Exclusion criteria

1. Age less than 18 years
2. Very large resections including partial resection of sacrum and patients considered for bilateral flap reconstruction
3. Large concomitant resection of vaginal wall where total (vaginal) wound closure is not an option
4. Expected survival less than one year at operation
5. Patient do not sign informed consent document, this group includes:
   1. Patients that do not want to participate and undergo allocation to treatment by randomization
   2. Patients or their legal representatives do not fully understand the role of participation

#### Violation of criteria for enrolment

The criteria for enrolment must be followed explicitly. If a patient who does not meet eligibility criteria is inadvertently enrolled, that patient should be discontinued from the study and the PI of the central study administration must be informed. See about discontinuations below.

The study aims to enrol a high proportion (>60 %) of eligible patients. Centres that fail to register patients operated on with either APE or EAPE and fail to enrol eligible patients will be discontinued from the study. The study administration claims the right to take part of data from participating centres revealing numbers of performed APE and EAPE operations during the inclusion phase.

### Disease diagnostic criteria

The diagnosis of rectal malignancy should be determined by histology before enrolment but absence of definitive histology is not an exclusion criterion if the decision of operation leans on sound clinical judgement. Primary cancer of rectum is defined as a malignancy that is not treated with curative intention before the operation. The definition allows preoperative oncologic treatments if the plan is to cure the patient with surgery. Recurrent cancer is defined as a cancer that develops after a previous treatment with curative intention. The previous treatment does not have to be surgical.

### Patient assignment

After patients give written informed consent and meet criteria for enrolment they may be randomised to one of the study arms before the operation if the extended APE procedure is decided and will be executed. If there is any doubt of the suitability of the procedure and the definitive decision of extended APE is done in the operating room, the randomization can be done intra-operatively. In these cases written informed consent must be given before the operation and the patient is informed that the assignment is decided later during the operation.

The randomization will be done on-line via the study homepage web application at [www.norrlandskirurgi.se](http://www.norrlandskirurgi.se/)

## Discontinuations

1. If a patient who does not meet eligibility criteria is inadvertently enrolled, that patient should be discontinued from the study.
2. If a centre has an unacceptably low proportion of registrations (entry) and/or enrolments there is a substantial risk for selection bias of study patients and the centre with its patients will be discontinued from the study.
3. Inevitable discontinuations, as if patients decease or are lost to follow up are registered by site investigator via the web application.
4. The patients are allowed to discontinue from the study without any reason if they wish according to ethical standards. If a patient chooses to discontinue from the study he/she should be asked if the study data collected until this point can be used or should it be erased.

All discontinuations should be registered via the study homepage web application at [www.norrlandskirurgi.se](http://www.norrlandskirurgi.se/) Result data from patients discontinued for reason 1-2 (above) will be erased and is not included in final analysis. Result data from patients discontinued for reason 3 (above) can be used until the date of discontinuation. Result data from patients discontinued for reason 4 (above) can be used until the date of discontinuation or all data will be erased depending on the patient’s decision.

## Surgical methods

### Preoperative measures

All patients should receive prophylactic antibiotic before the operation. One dose preoperatively is our recommendation but if the operation takes more than 4 hours to complete a second dose should be given within 24 hours from the first dose[13, 14]. If a 2nd generation cephalosporine is used alone, 2 additional doses every 8 hours after the first dose should be used [15]. Antithrombotic prophylaxis should be given with low molecular weight heparin and it should be continued 28 days[16-20]. Enhanced recovery programs may be used in whole or partially but the program or routines chosen by a clinic should not change during the study period. The patient should be informed about the postoperative rehabilitation programme by the physiotherapist. One of two available rehabilitation programmes should be chosen and used for all patients during the whole study period. See postoperative rehabilitation programmes below.

### First part of the operation

The following technique description is cited directly from the publication of Holm et al.[7] The abdominal part of the operation is performed as in conventional APR, with one important modification: the mesorectum is not dissected off the levator muscle. Thus the mobilization is stopped at the upper border of the coccyx posteriorly, just below the autonomic nerves laterally and anteriorly just below the vesicles in men or just below the cervix uteri in women. The divided left colon is brought out to form a colostomy and the abdomen is closed.

The patient is then turned into the prone jack-knife position with legs spread to enable the surgeon to stand between the legs with one assistant on each side. The anus is closed with a double purse-string suture. An incision is made around the anus and extended cranially to the lower part of the sacrum. The dissection continues in the subcutaneous fat, just outside the subcutaneous portion of the external anal sphincter. Following this plane the levator muscle is identified on both sides and the dissection is continued along the outer surface of the levator muscles proximally until the insertion on to the pelvic side wall. It is important to expose the levator muscles all around the circumference before entering the pelvis. The coccyx is then disarticulated from the sacrum and Waldeyer’s fascia divided. This permits entry into the pelvic cavity at the point where the intra-abdominal dissection stopped. The levator muscles are divided laterally on both sides, from posterior to anterior. The specimen is gently brought out and dissected off the prostate or the posterior vaginal wall. In the case of an anterior tumour, a portion of the prostate or the posterior vaginal wall may be resected en bloc. Finally, the remaining pelvic floor muscle fibres are divided just posterior to the transverse perineal muscles and the specimen is excised.[7]

N.B. In this study excision of the coccyx is optional. Omentoplasty to fill the small pelvis can also be performed according to the surgeon’s discretion. A suprapubic catheter to the urinary bladder should be placed during the laparotomy and kept until normal bladder emptying occurs. An intra-abdominal drain is positioned with the tip in the lesser pelvis and kept for 3-5 days but can be removed earlier if exudate volume is less than 50 ml/day.

### Reconstruction of pelvic floor with acellular porcine collagen implant (APCI)

A 10x10 cm or a 10x5 cm piece of the APCI (1.5 mm thick) is cut in the corners to fit the defect created in the floor of lesser pelvis. The implant is sutured in place with 2-0 polypropylene thread using interrupted sutures. Sutures attach to edge of sacrum or coccyx posteriorly in the wound, laterally to remnants or cut edges of levator ani muscle and anteriorly to capsule of prostate in men and to vaginal wall in female. It might be preferable to fold the implant anteriorly to produce a larger area of contact with the prostate or the vaginal wall. The wound is drained in two layers with the deep drain adjacent to the implant and the second superficially below skin. The two deep layers of the wound are closed with resorbable 2-0 thread and the skin with 3-0 monofilament thread.

Vaginal wall defects should be closed with absorbable sutures.

### Reconstruction of pelvic floor with gluteus maximus myocutaneous unilateral flap (GMF)

The following technique description is cited directly from the publication of Holm et al.[7]

The unilateral flap is usually based cranially with the length about 1·5: 1 in proportion to the base. At the lateral border of the base, a triangle of skin and fat is removed (Bürow’s triangle) in order to shorten the outer skin edge and to get it matched to the inner edge during the rotation. The design of the flaps is shown in Fig. 1. The lines for skin incision are drawn after removal of the specimen. Local anaesthetic with adrenaline is used to reduce bleeding and postoperative pain. The skin and subcutaneous tissue are incised down to the gluteus maximus where the fascia is also incised along the whole length of the wound to add mobility to the flap. About one-third to half of the muscle is then divided at its medial border, away from the hip joint capsule which should not be exposed. It is important to be aware of and to avoid the sciatic nerve that runs under the muscle. At the sub muscular level the dissection is extended in cranial and medial directions. The tissue layers are kept intact to avoid interruption of the perforating vessels. Mobility is tested continuously; as soon as the muscle part of the flap reaches the muscle on the other side of the defect without tension, the dissection is terminated. One of the two main vessels supplying the flap may occasionally be divided to attain sufficient mobility. The flap is sutured in four layers with interrupted sutures: in the muscle, in Scarpa’s fascia, in the deep dermis and in the skin. Two drains are placed and kept for 4–6 days, one deep to the muscle and one along the flap in the sub cutis. The wound is dressed with surgical tape.

Vaginal wall defects should be closed.

##


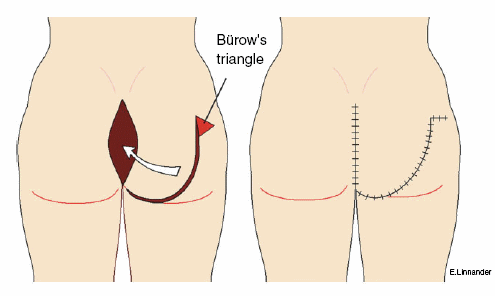


Figure 1. Design of unilateral myocutaneous flap. Figure from Holm et al. [7] with permission.

## Compliance

The study add time that is consumed for the follow-up procedures of involved patients. Patients may have to travel long distances for follow-up visits for the study. In the patient’s perspective this can be a positive experience provided that attempts are made to take care of the possible and actual problems that the patient may suffer. For this reason we recommend that all follow-up visits for the study are combined with visits to the attending surgeon and that may increase the compliance to follow the study plan.

## Blinding

Blinding in surgical studies is hard to accomplish. This study is performed without attempts for blinding and thus the attending surgeon, the examiner of physical performance and the patient do not have to be blinded.

## Concomitant therapy

Pre- and postoperative radiotherapy or chemo radiotherapy can be utilised according to multidisciplinary team decisions at each centre.

## Postoperative rehabilitation programmes

Due to already existing routines, we consider it not realistic to prescribe only one uniform programme. Instead, the patients will be allocated to either an established programme[7] or to an accelerated programme that has been used in another centre. Both rehabilitation programmes are standardized, written and should be followed explicitly. The descriptions of both programmes can be downloaded from the study webpage at [www.norrlandskirurgi.se](http://www.norrlandskirurgi.se/).

Participating centres must choose one of the rehabilitation programmes and use that program for all their study patients. The rehabilitation programmes at hand are studied earlier and considered safe [21].

## Evaluation of outcome measures and safety

### Measures of physical performance

The study physiotherapist or nurse will examine measures of physical performance.

A “timed-stands test” is to be performed by recording the time needed to stand ten times from a standard chair[9, 10]. The test gives a measure of lower extremity muscle strength, muscle coordination and tenderness of the gluteal region (caution when sitting) and gives the possibility to correlate the results to a reference population studied earlier[9]. The test is validated.

A complementary step test is performed at some participating centres (optional test)[22].

The study physiotherapist or nurse will process measures of quality of life, pain, discomfort and ability to sit. Data will be registered on forms (see Appendix) or directly in the electronic database.

Quality of life is measured with the global instrument EQ-5D and the cancer specific instrument EORTC QLQ-C30 and QLQ-CR29. The forms will be sent to the patient prior to the follow-up visits and the study physiotherapist or nurse who cares for the data to be registered in the study database will collect the completed forms. Completeness of data should be checked during the visit so that any incomplete data can be corrected together with the patient.

Pain in different postures will be evaluated with a 100 mm visual analogue scale (VAS). Discomfort and anatomical distribution of pain will be mapped with a specific figure or chart where patients can indicate areas of discomfort or pain.

The patients’ ability to sit will be evaluated during the initial interview by observation of how the patients sit in the chair during the first 10 minutes. The patients will not be told about the observation made by the examiner. Three categories of ability to sit will be used, a) normal sitting during 10 minutes, b) sitting with some kind of aid (cushion or ring) or compensating weight bearing with a not relaxed posture and c) not able to sit at all.

Optional tests for skin sensitivity in the perineal area (with von Freys filament) and pressure threshold for pain (with Algometer) at the sciatic tubercles will be measured at some centres.

### Measures of complications including the surgical and oncologic result

Measures of complications and the surgical as well as the oncologic result will be considered and registered **by the attending surgeon** during the follow-up visits. Postoperative complications will be classified according to Dindo-Clavien[12] and registered on a paper form (see Appendix) alternatively directly in the electronic database via the web application.

#### Definitions of surgical result and wound healing measures

The local surgical result in the perineal wound will be classified according to the Southampton Wound Assessment Scale[11] that is validated[23].

- 0 Normal healing
- I Normal healing with mild bruising or haematoma
- II Erythema plus other signs of inflammation
- III Clear or haemoserous discharge
- IV Pus
- V Deep or severe wound infection with or without tissue breakdown; haematoma requiring aspiration

The grade of the above wound healing classes is registered for each patient at the 3 month follow-up event and applies for the present status or for the postoperative period up to 3 months. The worst class noted during the follow-up should be recorded.

In addition, at all follow-up visits

a) persisting sinus of fistula in perineal wound and

b) removal of implant (only APCI) and

c) excision of a myocutaneous flap in part or whole (only GMF) and

d) occurrence of perineal hernia should be noted.

The healing of the laparotomy wound is not graded in this study but disturbances of laparotomy wound healing will be registered as an adverse event among other circumstances, see below.

#### Definitions of oncologic result

Local recurrence and/or metastatic disease are registered as soon as the attending surgeon has verified the existence of the complication. The diagnosis may be based on clinical examination, histology, radiological examination or a combination of investigations.

Information of local recurrence and metastatic disease will be registered in the postoperative follow-up form (see Appendix) at every follow-up visit.

### Safety measures, early postoperative complications, and other clinical adverse events

Postoperative complications will be registered in the Swedish Colorectal Cancer Registry via a web application (INCA) directly by the participating units within Sweden. The register collects data of postoperative complications occurring in the time intervals 1-30 days and from 31 days until five years after the index operation. This registration is routine for all patients treated for rectal cancer in Sweden and is not changed for patients participating in the study. Interpretation of data of postoperative complications can be done for the early period as soon as the registration of the operation is completed in the register. The delay of registration of the operation in the Swedish Colorectal Cancer Registry may vary between different units but should be done within two months. It is the attending surgeon who completes the register. The registration in the Swedish Colorectal Cancer Registry of complications occurring in the later period (31 days until 5 years) takes place after every follow-up visit and this data can be used as a safety measure during the study.

Participating units outside Sweden can not register early or late postoperative complications in the Swedish Colorectal Cancer Registry (INCA) but a similar registration form will be available via the study homepage web application ([www.norrlandskirurgi.se](http://www.norrlandskirurgi.se/)) for use of units outside Sweden.

Both the surgeon and the physiotherapists (and study nurses) involved in the follow-up examinations of patients should report every serious adverse event that is observed among enrolled patients later during the study. The reports of adverse events are made via the study homepage web application for units outside Sweden and in the Swedish Colorectal Cancer Registry (INCA) for units in Sweden. These reports are continuously collected and evaluated by the study administration, and if any safety concern with the study appears, an independent advisor is consulted.

Once per year an independent advisor that together with the PI has the authority to stop the study if serious unexpected safety issues can be observed will evaluate the collected data of clinical adverse events. If the independent advisor and the PI make a decision to stop the study, the decision must be written and motivated in detail and distributed to all participating centres without delay.

## Quality control and quality assurance

To ensure accurate and reliable data the study administration will do the following:

- Provide instructional material to the study sites, as appropriate (manual for physical examination etc.)
- Update study homepage at [www.norrlandskirurgi.se](http://www.norrlandskirurgi.se/) with relevant information and study material
- Start-up training, meeting with investigators
- Be available for consultation
- Conduct quality review of database (a monitor may be sent to participating centres)

# Data analysis methods

## Sample size

The sample size is calculated with the following assumptions:

- Estimated proportion of patients with low performance in TST with GMF 63%
- Estimated proportion of patients with low performance in TST with APCI 30%
- Estimated loss of data/cases 20% (mortality etc.)

The calculation gives that a total of 108 patients (54 patients in each study arm) are needed with 90% power and a significance level of 0.05 (alpha).

If the anticipated three-year inclusion phase of the study is too short for including 108 patients an alternative power calculation with 80% power will be used instead and the study will include a total of 80 patients only before the inclusion phase will be closed.

## Interpretation of noninferiority

The noninferiority margin for the study is set on the basis of a minimally important clinical effect, which is a subjective judgement of the best expertise available. This approach remains the only alternative since there are no historical placebo controlled trials in the field for guidance and for practical reasons such trials are not possible to conduct.

We consider that a minimally important clinical effect applies for this study if results differ less than 10%. The noninferiority margin is thus set to 10%. This means that the results from study arms are considered clinically having no important differences if the results from test operation (APCI) differ less than 10% in the main outcome. We claim noninferiority for test operation if the result will be in the range less than 10% inferior result up to a trend for superior result but not statistically significant for the main outcome. If the result shows a statistically significant difference we claim superiority (or inferiority) of the test operation. If we find noninferiority based on the main outcome we may still draw conclusions about which operation should be preferred, based on results from secondary outcomes.

## Data to be analyzed

All primary analyses in this study will be performed according to the intent-to-treat (ITT) principle, that is, patients will be allocated to treatment groups corresponding to their assigned treatment, even if the patient does not receive the correct treatment.

The intention-to-treat population will consist of all randomised patients who are operated with EAPE.

Per-protocol analyses are secondary and may be used if special circumstances motivate to use such. If noninferiority is to be stated we will do a per-protocol analysis in addition to the ITT analysis and if both analyses are non significant it will give more evidence of noninferiority. The per-protocol population will consist of all patients in the intent-to-treat population who have met all protocol requirements and who have successfully completed the trial up till one year.

### Patient disposition

Reasons for discontinuations in the study will be compared between the 2 treatment groups. Result tables or the Consort[24] diagram will reveal the number and proportion of patients who have completed the study as well as patients that have discontinued, grouped by reason for discontinuation.

### Patient characteristics

Age, gender, primary or recurrent rectal cancer will be registered when the patient is randomised via the web application and will be included in the summary reports.

Preoperative oncologic treatment and pre- and postoperative tumour classification (cTNM and pTNM) will be registered in (and retrieved from) the Swedish Colorectal Cancer Registry for Swedish patients and for patients outside Sweden a similar form with TNM data is completed via the study homepage web application by the local investigator. The pTNM classification will also be included in the summary reports.

### Primary analysis

Performance in Timed-stands test (TST) measured in seconds (time) will be compared between study arms at 6 months from the operation and tested with appropriate statistical tests. The proportion of patients that perform worse than the upper limit of reference values may be used as a parameter for comparison. TST reflects physical performance that is the primary outcome of the study.

### Secondary analyses

1. The change of physical performance, i.e. the difference between preoperative and postoperative results in TST at 3, 6 and 12 months will be compared between study arms.
2. Primary wound healing at 3 months is categorized in subclasses (SWAS[11]) and the proportions of patients in different classes are compared between study arms.
3. Complications are categorized according to classification by Dindo-Clavien [12] and the proportions of patients in different classes are compared between study arms at 3, 6 and 12 months.
4. Ability to sit is defined in three classes and the proportions of patients in different classes are compared between study arms at 3, 6 and 12 months.
5. Pain and discomfort in gluteal region measured with VAS and analyzed with appropriate statistical methods. Comparisons will be done at 3, 6 and 12 months.
6. Quality of life is measured with EQ-5D and EORTC forms C30 and CR29 and the individual responses are calculated to a health utility index using an appropriate table/tariff for the general population[25, 26] Spot measures at 3, 6 and 12 months and differences between pre-treatment and postoperative measurements will be compared.
7. The proportion of patients that present local recurrence during the study period will be compared between study arms.

### Subgroup analyses

1. Costs of surgical treatments and QALYs gained are calculated for a subpopulation of the study population. Registration of parameters needed for the health economic calculations is optional for the participating centres and the subpopulation will be included only from centres that collect health economic data in their computer systems. Costs of surgical treatments including surgical complications up till 12 months will be calculated according to health economic standards and QALYs gained will be compared between study arms.
2. Two different rehabilitation programmes will be used for study patients and every participating centre must decide which of the programmes to use. The centre must stay to the chosen rehabilitation programme throughout the study. A subgroup analysis will be performed with the aim to reveal if the rehabilitation program has an impact on the main and secondary outcomes.

### Safety analyses

An independent observer will be appointed with the task to contact each centre yearly and ask for unforeseen adverse events and problems. If severe adverse events occur the PI and the independent observer have the responsibility to decide whether the study can continue or should be stopped.

All safety analyses comparing the treatment groups will be performed based on the intent-to-treat population. All statistical tests of safety will be conducted at a two-sided alpha-level of 0.05. The safety analysis variables include, but are not restricted to:

- Postoperative mortality in 30 days
- Postoperative complications Grade IIIb or higher according to Dindo-Clavien
- Proportions of serious adverse events

### Interim analyses

Interim analyses will be performed only if the study inclusion period exceeds 5 years. Safety analyses are exceptions that are performed whenever proposed by the independent observer.

# Informed consent, ethical review and regulatory considerations

## Informed consent

The informed consent document with study information will be used to explain in simple terms to patients what participation in the study means for the patient. The patient will get information of the risks, benefits and the alternatives available.

It is the investigators responsibility to see that informed consent is obtained from each patient or legal representative before enrolment in the study. The informed consent document must be signed and dated and finally stored in archive at each participating centre. The signed document may also be photo-scanned to an electronic document and included in the hospital electronic patient file. Once the scan copy is secured to an electronic patient file the paper document may be destroyed.

## Ethical review

The PI will apply for ethical board review at the Regional Ethical Review Board at Umeå University. The application will cover all study sites in Sweden but study sites in other countries may have to apply separately according to the local regulations. It is the site investigators responsibility in other countries than Sweden to apply and hold the local ethical review board approval for the study. After the Swedish application has been approved, the PI will provide site investigators with documentation of ethical review board approval of the protocol and informed consent document including patient information.

## Regulatory considerations

This study will be conducted in accordance with the ethical principles stated in the most recent version of the declaration of Helsinki or the applicable guidelines on good clinical practice, whichever represents the greater protection of the individual.

After reading the protocol, each site investigator will sign the protocol signature page, see Appendix last page, and return it to the PI.

# References

1. Miles, W.E., *A method of performing abdominoperineal excision for carcinoma of the rectum and of the terminal portion of the pelvic colon.* Lancet, 1908. **2**: p. 1812-1813.

2. Davies, M., et al., *Local recurrence after abdomino-perineal resection.* Colorectal Dis, 2009. **11**(1): p. 39-43.

3. Heald, R.J., et al., *Abdominoperineal excision of the rectum--an endangered operation. Norman Nigro Lectureship.* Dis Colon Rectum, 1997. **40**(7): p. 747-51.

4. Thirion, P., et al., *Survival impact of chemotherapy in patients with colorectal metastases confined to the liver: a re-analysis of 1458 non-operable patients randomised in 22 trials and 4 meta-analyses. Meta-Analysis Group in Cancer.* Ann Oncol, 1999. **10**(11): p. 1317-20.

5. Marr, R., et al., *The modern abdominoperineal excision: the next challenge after total mesorectal excision.* Ann Surg, 2005. **242**(1): p. 74-82.

6. Miles, W.E., *The radical abdominoperineal operation for cancer of the pelvic colon.* BMJ, 1910: p. 941-943.

7. Holm, T., et al., *Extended abdominoperineal resection with gluteus maximus flap reconstruction of the pelvic floor for rectal cancer.* Br J Surg, 2007. **94**(2): p. 232-8.

8. Edwards, T., et al., *Porcine collagen (Permacol) reconstruction following prone abdomino-perineal excision.* Colorectal Dis, 2009. **11**(Suppl II): p. 12.

9. Csuka, M. and D.J. McCarty, *Simple method for measurement of lower extremity muscle strength.* Am J Med, 1985. **78**(1): p. 77-81.

10. Newcomer, K.L., H.E. Krug, and M.L. Mahowald, *Validity and reliability of the timed-stands test for patients with rheumatoid arthritis and other chronic diseases.* J Rheumatol, 1993. **20**(1): p. 21-7.

11. Bailey, I.S., et al., *Community surveillance of complications after hernia surgery.* Bmj, 1992. **304**(6825): p. 469-71.

12. Dindo, D., N. Demartines, and P.A. Clavien, *Classification of surgical complications: a new proposal with evaluation in a cohort of 6336 patients and results of a survey.* Ann Surg, 2004. **240**(2): p. 205-13.

13. Lohsiriwat, V. and D. Lohsiriwat, *Antibiotic prophylaxis and incisional surgical site infection following colorectal cancer surgery: an analysis of 330 cases.* J Med Assoc Thai, 2009. **92**(1): p. 12-6.

14. Suehiro, T., et al., *Prolonged antibiotic prophylaxis longer than 24 hours does not decrease surgical site infection after elective gastric and colorectal surgery.* Hepatogastroenterology, 2008. **55**(86-87): p. 1636-9.

15. Fujita, S., et al., *Randomized, multicenter trial of antibiotic prophylaxis in elective colorectal surgery: single dose vs 3 doses of a second-generation cephalosporin without metronidazole and oral antibiotics.* Arch Surg, 2007. **142**(7): p. 657-61.

16. Rasmussen, M.S., *Preventing thromboembolic complications in cancer patients after surgery: a role for prolonged thromboprophylaxis.* Cancer Treat Rev, 2002. **28**(3): p. 141-4.

17. Bergqvist, D., et al., *Duration of prophylaxis against venous thromboembolism with enoxaparin after surgery for cancer.* N Engl J Med, 2002. **346**(13): p. 975-80.

18. Osborne, N.H., T.W. Wakefield, and P.K. Henke, *Venous thromboembolism in cancer patients undergoing major surgery.* Ann Surg Oncol, 2008. **15**(12): p. 3567-78.

19. Bergqvist, D., *Venous thromboembolism: a review of risk and prevention in colorectal surgery patients.* Dis Colon Rectum, 2006. **49**(10): p. 1620-8.

20. Geerts, W.H., et al., *Prevention of venous thromboembolism: the Seventh ACCP Conference on Antithrombotic and Thrombolytic Therapy.* Chest, 2004. **126**(3 Suppl): p. 338S-400S.

21. Haapamaki, M., et al., *Physical performance and quality of life after extended abdominoperineal excision of rectum and reconstruction of the pelvic floor with gluteus maximus flap.* Dis Colon Rectum, 2010. **In press**.

22. Frändin, K. and G. Grimby, *Assessment of physical activity, fitness and performance in 76-year-olds.* Scand J Med Sci Sports, 1994. **4**: p. 41-46.

23. Karran, S.J., et al., *Antibiotic prophylaxis in clean surgical cases and the role of community surveillance.* Eur J Surg Suppl, 1992(567): p. 31-2.

24. Moher, D., K.F. Schulz, and D.G. Altman, *The CONSORT statement: revised recommendations for improving the quality of reports of parallel-group randomised trials.* Lancet, 2001. **357**(9263): p. 1191-4.

25. Ohinmaa, A.E.H. and H. Sintonen. *Modeling EuroQol values of Finnish adult population*. in *EuroQol Plenary Meeting*. 1995. Barcelona: Institut Universitari de Salut Publica de Catalunya.

26. Oppe, M., A. Szende, and F. de Charro, *Comparative review of Visual Analogue Scale value sets*, in *EQ-5D Value Sets: Inventory, Comparative Review and User Guide*, A. Szende, M. Oppe, and N. Devlin, Editors. 2007, Springer: Dordrecht. p. 29-38.

# Appendix

### **Form S1:** **Surgeon, first postoperative visit, workup and TNM**

(same as Swedish CRC register at [www.incanet.se](http://www.incanet.se/), paper form or [www.norrlandskirurgi.se](http://www.norrlandskirurgi.se) outside Sweden)

SEPARATE DOWNLOAD

### **Form S2**: **Surgeon, early complications within 30 d**

(same as Swedish CRC register at [www.incanet.se](http://www.incanet.se/), paper form or [www.norrlandskirurgi](http://www.norrlandskirurgi) outside Sweden)

SEPARATE DOWNLOAD

### **Form S3: Surgeon, all postoperative visits**

SEPARATE DOWNLOAD

### **Form S4: Surgeon, late complications, postoperative visits later than 30 days**

(same as Swedish CRC register at [www.incanet.se](http://www.incanet.se/), paper form or [www.norrlandskirurgi.se](http://www.norrlandskirurgi.se) outside Sweden)

SEPARATE DOWNLOAD

### **Form P1: Physiotherapist, pre-treatment**

**Inskrivningsformulär (ifylls av sjukgymnast/ studiesjuksköterska)**

SEPARATE DOWNLOAD

### **Form P2: Physiotherapist, test form**

NEAPE Bedömningsformulär för fysisk funktion (sjukgymnast/studiesköterska)

🞏 preoperativt 🞏 3 mån 🞏 6 mån 🞏 12 månader

SEPARATE DOWNLOAD

### Form P3: Physiotherapist or study nurse, postoperative

### NEAPE uppgifter om RT och OP

Fylls i senast 3 månader efter operation.

SEPARATE DOWNLOAD

QoL formulär EQ-5D och EORTC C30 och CR29 på följande sidor

SEPARATE DOWNLOAD

Patientinformation och informerat samtycke till NEAPE studien

Du tillfrågas om deltagande i en forskningsstudie om två olika sätt att försluta såret i samband med operation för cancer i ändtarmen. Flera sjukhus i norden är med i studien.

Två alternativa metoder för rekonstruktion av bäckenbotten har etablerats vid operation av lokalt avancerad lågt sittande rektalcancer. Man kan rekonstruera vävnadsdefekten som uppstår vid canceroperationen med en hudmuskellambå, eller med ett inplantat (kollagenplatta) som täcks med hud och underhudsfett från sårkanten. I tekniken med hudmuskellambå friar man en del av sätesmuskeln och ovanpåliggande hud som flyttas mot mitten för att täcka defekten medan man med inplantattekniken överbryggar defekten med en kollagenplatta och friar lite hud och underhudsfett från sårkanterna så att såret kan förslutas. Inga jämförande studier har gjorts mellan dessa tekniker och således saknas vetenskapligt underlag för att bestämma vilken operationsmetod som borde användas för största patientnytta. Läget av operationssåret i sätesregionen gör att rekonstruktionen har betydelse för patientens rörelseförmåga, sittförmåga och smärtupplevelse efter canceroperationen.

Vi avser att jämföra resultatet av de två etablerade operationsmetoderna med avseende på fysisk förmåga som primär frågeställning. Andra frågeställningar är vilken metod som ger bättre sårläkning, bättre förmåga att sitta, mindre postoperativ smärta, bättre livskvalitet och mindre kirurgiska komplikationer.

Patienter som skall opereras för rektalcancer och som deltar i studien, kommer slumpmässigt att fördelas till en av två metoder för rekonstruktion av defekten i bäckenbotten som uppstår vid respektive operation. I studien ingår besök och uppföljning hos sjukgymnast eller studiesjuksköterska och då ställs ett antal frågor och vissa tester på fysisk förmåga kommer att göras före respektive efter operationen. Dessa undersökningar tar ungefär en timme per gång.

Denna studie har godkänts av Etikprövningsnämnden i Umeå. Deltagandet i studien är helt frivilligt och kan när som helst avbrytas utan att skäl för detta behöver anges. Eventuellt avbrytande av studien kommer inte att påverka Din fortsatta handläggning. Om Du väljer att avbryta Ditt deltagande i studien kommer Du att tillfrågas om vi i vår slutanalys får använda det data som Du från studiestart fram till avbrytandet bidragit med. Vid sammanställning och vid offentliggörande av resultat kommer ingen patient att kunna identifieras.

Personuppgiftsansvarig är Västerbottens läns landsting. Allt forskningsdata behandlas i enlighet med personuppgiftslagen och Du har rättighet att få ett utdrag med Dina egna uppgifter ur registret om Du skriftligen begär detta av kontaktpersonen. Huvudman för studien är Kirurgcentrum vid Norrlands Universitetssjukhus. Om Du ej önskar deltaga i studien kommer du att opereras med den operationsmetod Du och din läkare kommer överens om. Önskar Du ytterligare information eller har frågor, kontakta studieansvariga läkare på sjukhuset där du blir opererad.

Sjukhus: Studieansvarig läkare:

Telefon: E-post:

(Ovanstående uppgifter ifylles av lokalt studieansvarig läkare på respektive deltagande sjukhus.)

Läkare som delgivit Dig information om NEAPE studien:

………………………………………………..

## Informerat samtycke

Jag har muntligen informerats om NEAPE studien och tagit del av skriftlig information. Jag är medveten om att mitt deltagande är frivilligt och när som helst kan avbrytas utan att skäl behöver anges.

………………………………………………... (Ort och datum)

……………………………………………….. (underskrift)

……………………………………………….. (personnummer)

(Denna sida kan skannas och bifogas elektronisk patientjournal alternativt arkiveras med pappersjournal på respektive sjukhus.)

## Protocol signature

I confirm that I have read this protocol and understand it. I will work according to this protocol and to the ethical principles of the Declaration of Helsinki and according to guidelines for good clinical practice.

I will accept the PI’s and eventual monitor’s overseeing of the study and I accept that the number of patients operated on with abdominoperineal excision (extended or not) at my institution/hospital/department during the study period will be revealed when requested by the PI or other representative of the central study administration.

I will accept that my name and affiliation is public and mentioned as site investigator at clinicaltrials.gov web registry for clinical trials.

If I am the site investigator at a hospital not in Sweden, I will promptly submit the protocol to an applicable ethical review board.

______________________________ ___________________

Signature of investigator Date

______________________________ ___________________

Investigator printed name E-mail

______________________________ ___________________

Investigator Title Office phone

________________________________________________________________

Name and address of facility

Write your preferred username (minimum 6 characters) and password (minimum 9 characters) for login to randomisation page.

Username:___________________ Password:____________________

***Copy for own filing and send the completed Protocol Signature to:***

Markku Haapamäki, MD, PhD,

Department of surgery and perioperative sciences

Umeå University

SE-901 85 Umeå

Sweden

PI contact: Phone: +46-90-785 2013

Fax: +46-90-785 1156

E-mail: markku.haapamaki@surgery.umu.se

## Amendment History / Change Log

**Protocol title:**
Nordic Extended Abdominoperineal Excision (NEAPE) study

**Original protocol version:** Version 16, dated 13 September 2012
**Amended based on:** Published protocol in BMJ Open 2019
**Nature of amendment:** Methodological clarification and refinement
**Impact on participants:** None
**Impact on study conduct:** None
**Impact on primary objective or endpoints:** None

### Description of changes

**1. Sample size and power calculation (Clarification and update)**
The sample size and power calculation section has been updated to reflect a refined and more conservative statistical approach as described in the subsequently published study protocol.

- In the original protocol (Version 16), the sample size calculation was based on an assumed proportion of low physical performance (Timed‑Stands Test) of **63% in the GMF group and 30% in the APCI group**, with an anticipated **20% loss to follow‑up**, resulting in a required sample size of **108 patients (54 per group)** for 90% power at a two‑sided alpha of 0.05.
- In the updated protocol version, the sample size calculation is based on revised assumptions:
  - Estimated proportion of low physical performance in the control group (GMF): **63%**
  - Estimated proportion of low physical performance in the experimental group (APCI): **40%**
  - **Non‑inferiority margin: 10%**
  - **Anticipated attrition: 10%**
- Based on these assumptions, **76 evaluable patients (38 per group)** are required to achieve **90% statistical power** at a significance level of **5%**, and thus a total of **85 patients** are planned to account for attrition.

The revision reflects updated empirical data and improved methodological consistency with a non‑inferiority framework.

**2. No other protocol changes**
No other sections of the protocol have been modified. Specifically:

- Study objectives, hypotheses, endpoints, inclusion and exclusion criteria, randomisation procedures, interventions, follow‑up schedule, outcome assessments, and ethical considerations remain unchanged.
- The amendment does not alter the risk–benefit profile for study participants.

### Rationale for amendment

This amendment was introduced to ensure that the study protocol accurately reflects the refined statistical assumptions and sample size calculation used in the peer‑reviewed and publicly available protocol publication, thereby improving transparency, methodological rigor, and consistency between protocol versions.
